# Infection of Human Cells by SARS-CoV-2 and Molecular Overview of Gastrointestinal, Neurological, and Hepatic Problems in COVID-19 Patients

**DOI:** 10.3390/jcm10214802

**Published:** 2021-10-20

**Authors:** Mahdie Rahban, Agata Stanek, Amirreza Hooshmand, Yasaman Khamineh, Salma Ahi, Syed Naqui Kazim, Faizan Ahmad, Vladimir Muronetz, Mohamed Samy Abousenna, Samaneh Zolghadri, Ali A. Saboury

**Affiliations:** 1 Institute of Biochemistry and Biophysics, University of Tehran, Tehran 1417614335, Iran; mrohban@ut.ac.ir; 2 Department of Internal Medicine, Angiology and Physical Medicine, Faculty of Medical Sciences in Zabrze, Medical University of Silesia, Batorego 15 St., 41-902 Bytom, Poland; astanek@tlen.pl; 3 Young Researchers and Elite Club, Jahrom Branch, Islamic Azad University, Jahrom 7414785318, Iran; amirrezahoushmand66@gmail.com (A.H.); yasamankhamineh@yahoo.com (Y.K.); 4 Research Center for Noncommunicable Diseases, Jahrom University of Medical Sciences, Jahrom 7414846199, Iran; salmaahi.61@gmail.com; 5 Centre for Interdisciplinary Research in Basic Sciences, Jamia Millia Islamia, New Delhi 110025, India; skazim@jmi.ac.in (S.N.K.); faizan.ahmad.jmi@gmail.com (F.A.); 6 Belozersky Institute of Physico-Chemical Biology, Lomonosov Moscow State University, 119234 Moscow, Russia; vimuronets@mail.ru; 7 Central Laboratory for Evaluation of Veterinary Biologics, Agriculture Research Center, Cairo 11517, Egypt; mohamedsamy2020@hotmail.com; 8 Department of Biology, Jahrom Branch, Islamic Azad University, Jahrom 7414785318, Iran

**Keywords:** gut flora, neurological symptoms, coronavirus disease 19, angiotensin-converting enzyme-2, spike protein, hepatic derangements

## Abstract

The gastrointestinal tract is the body’s largest interface between the host and the external environment. People infected with SARS-CoV-2 are at higher risk of microbiome alterations and severe diseases. Recent evidence has suggested that the pathophysiological and molecular mechanisms associated with gastrointestinal complicity in SARS-CoV-2 infection could be explained by the role of angiotensin-converting enzyme-2 (ACE2) cell receptors. These receptors are overexpressed in the gut lining, leading to a high intestinal permeability to foreign pathogens. It is believed that SARS-CoV-2 has a lesser likelihood of causing liver infection because of the diminished expression of ACE2 in liver cells. Interestingly, an interconnection between the lungs, brain, and gastrointestinal tract during severe COVID-19 has been mentioned. We hope that this review on the molecular mechanisms related to the gastrointestinal disorders as well as neurological and hepatic manifestations experienced by COVID-19 patients will help scientists to find a convenient solution for this and other pandemic events.

## 1. Introduction

Coronavirus disease 2019 (COVID-19) is an infectious respiratory disease caused by a new coronavirus—namely, SARS-CoV-2 (severe acute respiratory syndrome coronavirus 2) [1]. As of 23 August 2021 [2], it has infected over 211 millions of people in more than 190 countries and caused more than 4.7 million deaths all over the world. In order to control and manage the COVID-19 pandemic and avoid the recurrence of future pandemics, the world’s scientists have attempted to find appropriate solutions through interdisciplinary cooperation [3]. During the first decade of the 21st century, development in life sciences has provided us with a deep understanding of the interactions between pathogens and their hosts. For example, there have been remarkable innovations in molecular biology in discovering pathogenesis mechanisms and diagnosis techniques, as well as advances in drug discovery, vaccines, and computational simulation due to the occurrence of various epidemics [4,5]. 

Many researchers have presented valuable information on the underlying events of SARS-CoV-2 infection and its related symptoms. Although COVID-19 is mainly a pulmonary disease, gastrointestinal symptoms have also been reported in some patients with this virus [6]. Respiratory symptoms, fever, and cough, as the main clinical signs among COVID-19 patients, have been investigated more than other symptoms, and general agreement about others, including the outbreak of gastrointestinal (GI) symptoms, is lacking [7].

Results indicate that the SARS-CoV-2 can damage the gastrointestinal system directly or indirectly through inflammatory reactions and may lead to several digestive problems, including vomiting, diarrhea, nausea, diminished appetite, and abdominal pain in patients with COVID-19 [8,9]. It has been determined that these gastrointestinal symptoms along with intestinal flora dysbiosis occur due to SARS-CoV-2 attacking the digestive tract of the host. Based on some reports, it seems that individuals with gastrointestinal problems are more likely to experience severe COVID-19 disease, which may be seen as a predictor of the development of severe respiratory disorders [10,11]. Additionally, several reports and pieces of evidence suggest that SARS-CoV-2 most likely either directly or indirectly affects the enteric nervous system, leading to gut dysfunction and neuro-gastrointestinal manifestations [12]. 

The hepatic consequences of SARS-CoV-2 infection are an important problematic component of COVID-19 that is most important in patients with earlier liver disease who are at remarkably high risk of severe COVID-19 and death [13,14,15]. Although the entire impact of COVID-19 on the liver is not clear, biochemical abnormalities in the liver are occurring in approximately 15–65% of people infected with SARS-CoV-2. Some reports have demonstrated that an elevation in serum liver enzymes is associated with adverse outcomes such as shock, intensive care unit (ICU) admission, and mechanical ventilation. These findings were made by Marjot et al. [16] in a review article. Additionally, the possible underlying direct and indirect mechanisms of liver injury have also been discussed.

In another review article published by Jakubiak et al., the gastrointestinal symptoms and pathophysiology of gastrointestinal dysfunction and probable mechanisms of liver injury in the course of COVID-19 were summarized and discussed [17]. In this study, we aimed to provide an overview of neuro-gastrointestinal and hepatic manifestations, discuss the potential cellular and molecular mechanisms involved, and provide useful information for finding appropriate management and treatment solutions for COVID-19.

## 2. Gastrointestinal Pathogenesis of SARS-CoV-2

Based on the reports, SARS-CoV-2 shares common clinical and epidemiological features with SARS-CoV and MERS CoV (Middle East Respiratory Syndrome coronavirus) due to its 70% and 40% similarity, respectively, in genetic sequence [18]. It is well established that angiotensin converting enzyme-2 (ACE2), a relevant player in the renin-angiotensin system (RAS), is the key receptor for SARS-CoV-2 invasion and the infection of human target cells that is similar to SARS-CoV. ACE2 converts angiotensin-2 to angiotensin-(1–7), which negatively regulates the active RAS [19]. Previous investigations of SARS-CoV have shown that its cellular entry occurs via the epithelial ACE2 receptor [20]. For this purpose, the receptor-binding domain (RBD) of viral surface glycoproteins named the spike (S) protein binds to the ACE2 receptor with a high affinity and thus releases the virus to the cell [21]. ACE2, a type I transmembrane glycoprotein, consists of a longer extracellular area with carboxypeptidase activity and a short intracellular cytoplasmic tail [22].

The trimeric spike (S) glycoproteins on the viral surface are composed of about 1300 amino acid residues and contain three main parts: the extracellular domain, the transmembrane domain, and the intracellular tail. The spike protein is cleaved by host cell transmembrane serine protease 2 (TMPRSS2) into S1 (the peripheral fragment) and S2 (membrane-spanning fragment) subunits. The S1 subunit contains a receptor-binding domain with several disordered regions, facilitating interactions with host cell receptors, whereas the S2 subunit fuses with the host cell membrane and releases the viral RNA for replication and translation by the host cell machinery [23,24,25].

The interaction of ACE-2 with spike proteins has been presented previously in a comprehensive review. Additionally, the small molecular inhibitors that can limit these interactions and be used as potential therapeutic platforms have been discussed [26].

Researchers have employed single-cell sequencing to identify the amount of ACE2 expressed in several organs. The obtained results show that ACE2 was largely found not only in the lung tissue but that it is also overexpressed in the gastrointestinal tract, including the esophagus and the absorptive intestinal epithelium in the colon and ileum. The ACE2 expression in the gastrointestinal tract, particularly in the colon, is approximately 100-fold higher than that in the alveolar cells of the lungs and respiratory system [27,28]. 

In another study, results from ACE2 immunohistochemical staining showed that the liver and digestive organs have higher ACE2 expression levels compared to the respiratory system. However, in lung tissue, the ACE2 expression level increased with age, which might explain, to some extent, why elderly people with COVID-19 are more likely to develop pneumonia. Recently, research on the pathophysiology of COVID-19 has indicated that the pattern and level of human ACE2 enzyme expression in different tissues might be correlated with the various different symptoms and outcomes of COVID-19 [29]. The abundance of this receptor in the epithelium of the GI tract and digestive system, especially in the small and large intestines, makes them susceptible to virus entry.

Some symptoms in patients with COVID-19, such as cardiovascular, kidney, GI, and brain manifestations, are associated with the co-expression of ACE2 and TMPRSS2. The intestinal brush border cells have high levels of TMPRSS2, transmembrane serine protease 2, which is capable of robust and persistent infection. These cells would be a useful tool for understanding the pathogenesis of SARS-CoV-2 in the GI tract [14]. TMPRSS2 and ACE-2 co-expression in GI could be the entry route for SARS-CoV-2 virus in absorptive enterocytes of the colon and ileum, resulting in further damage to the mucous membrane barrier, the development of inflammatory cytokine production, and the GI symptoms associated with COVID-19. Expression of ACE2 can be altered by several factors, including GI malignancies, which could also increase the severity of COVID-19 infection due to higher expression of ACE-2 and TMPRSS2 [30]. These findings suggest that a high expression of ACE2 in the digestive system may be a potential route of infection. Therefore, in COVID-19 patients, understanding the mechanism of damage to the digestive tract is very important in order to guide clinical treatment.

Perisetti et al. have reported that people with hypochlorhydria are more sensitive to viral infections due to a higher viral load entering the small intestine via ACE2 [31]. Sung et al. concluded that the down regulation of ACE2 by SARS-CoV-2 changes the uptake of certain amino acids; disrupts the gut barrier; increases the lipopolysaccharide and peptidoglycan levels of bacteria; and promotes systemic inflammation, contributing to the occurrence of a cytokine storm [32]. Based on these findings, the mechanism correlated with GI tract manifestations in SARS-CoV-2 infection could be further studied through the mediation of ACE2 receptors. 

### 2.1. Microbiome Alterations, a Putative Mechanism of Gastrointestinal Manifestations in COVID-19 Patients

Normal intestinal flora (referring to the collective genomes of microorganisms including bacteria, fungi, viruses, etc.) plays a crucial role in maintaining the homeostasis of the immune system and the metabolic function and health of the host gastrointestinal tract. Normal human gut flora harbors trillions of microbes that play important roles in human biology and disease. A change in the most abundant genera of the intestinal flora may result in some pathological disorders, such as GI dysfunction, including diarrhea, nausea, and vomiting; inflammatory bowel disease (IBD); and neurodegenerative problems. According to Martinez-Guryn et al., much about this topic remains to be revealed, including distinguishing healthy versus unhealthy gut microbiomes, which are likely to be characterized by the host, pathogens, and environmental factors [33].

It has been shown that COVID-19 infection increases the risk of gut flora changes in patients. Therefore, it should be expected that the digestive disorders and the immune homeostasis alterations induced by the virus might be mediated, to some extent, by the gut flora [34,35]. Some patients with COVID-19 have shown a gut microbial imbalance, with low levels of *Bifidobacterium* and *Lactobacillus* [36]. It has been reported that the amounts of *Clostridium hathewayi, Coprobacillus*, and *Clostridium ramosum,* present are closely correlated with the severity of COVID-19 and inversely correlated with *Faecalibacterium prausnitzii* [37]. Furthermore, antimicrobials significantly alter the gut microbiota pattern, resulting in *Clostridioides difficile* infection as a consequence of bacterial disturbances in the gut. The gut microbiota plays an essential role in opposing the colonization of *Clostridioides difficile*, and this microbial perturbation creates a favorable environment for colonization and *Clostridioides difficile* infection [38,39]. Therefore, clinicians should be aware of the disadvantages of the extensive use of antibiotics and, consequently, possible *Clostridioides difficile* infection with SARS-CoV-2 co-infection.

Generally, several key factors in microbiome alterations in people infected with SARS-CoV-2 have been highlighted by Perisetti et al. [31]. The first is the increase in the release of pro-inflammatory cytokines. The second is antimicrobial medications, including antibiotics and antivirals. The third is changes in the lung flora and the ratio of pathogenic organisms. The fourth is enteral nutrition. Additionally, the fifth is aberrant mTOR (mechanistic Target of Rapamycin Kinase) activity [31]. All of these mechanisms are only potential, and there is no credible proof of whether they act separately or in collaboration with each other in the progression of digestive symptoms in COVID-19 patients [31]. Some researchers have suggested that the targeting of the intestinal flora could potentially be a useful strategy with which to fight SARS-CoV-2 infection [34].

#### 2.1.1. Pro-Inflammatory Cytokines

It is well known that patients infected with SARS-CoV-2 show increased levels of cytokines and inflammation biomarkers [31]. In fact, COVID-19 disease leads to an excessive activated immune response, with the uncontrolled production and release of a number of cytokines. This phenomenon, known as a cytokine storm, is the main reason for worsening of the condition of COVID-19 patients, including those with gastrointestinal disorders, which alters the gut motility and GI flora. A cytokine—namely, granulocyte monocyte colony-stimulating factor (GM-CSF)—is made up of different types of cells, such as macrophages, T cells, fibroblasts, and endothelial cells, and is heavily loaded in the gastrointestinal tract [40]. 

**Figure 1:**
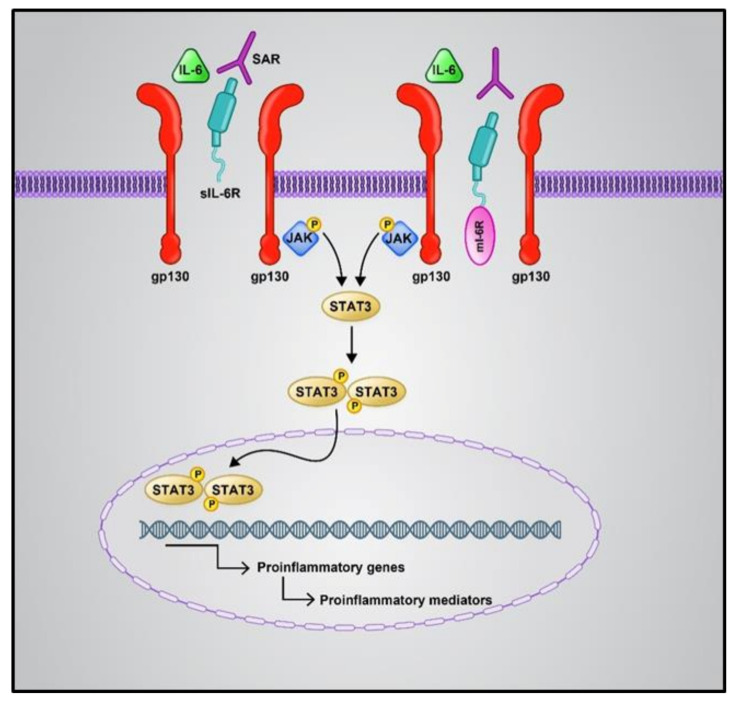
Cis and trans IL6 signaling pathways. The cis pathway is mediated by mIL6R as an anti-inflammatory, while the trans-signaling pathway is mediated by sIL6-R as a pro-inflammatory. Both the IL-6 signaling pathways converge in the activation of the JAK/STAT pathway. Phosphorylation of the receptor associated JAK1 is induced by the binding of numerous cytokines or growth factors to their specific receptors; then, STAT3 phosphorylation occurs via JAK1. Phosphorylated STAT3 is dimerized and transferred to the nucleus, which causes the expression of target genes contributing to angiogenesis, proliferation, immunosuppression, and inflammation; IL6: Interlukine 6, mIL6R: membrane form of IL-6 receptor, sIL6-R: soluble form of IL-6 receptor, JAK: Janus kinase, STAT: signal transducer and activator of transcription.

The first line of host defense against attacking pathogens relies on pattern recognition receptor (PRR)-mediated signaling (mostly Toll-like receptors) [41]. RNA release is recognized by RIG-I (viral RNA receptor retinoic-acid inducible gene I), cytosolic MDA5 receptor (melanoma differentiation-associated gene5), STING (stimulator of interferon genes), and cGA Snucleotidyltransferase (cyclic GMP-AMP synthase). This leads to the activation of downstream signaling, including pro-inflammatory factors (e.g., IL-6), antiviral cytokines, NF-*κ*B (nuclear factor-*κ*B), and IFN (interferon) [42,43]. COVID-19 patients show a suppressed Nrf2 (nuclear factor erythroid 2-related factor 2) pathway. Nrf2 is a cytoprotective factor that inhibits NF-κB and inhibits the expression of the inflammatory cytokines in macrophages in SARS-CoV-2-infected cells. Nrf2 reduces the ACE2 receptor expression in respiratory epithelial cells. Nrf2 activation plays a role in the execution of inflammation and decreases the intensity of cytokine storms [44]. Additionally, high levels of IL-6 play a key role in worsening cytokine storms, caused by respiratory failure and acute respiratory distress syndrome. IL-6 mainly makes use of two pathways: *cis* and *trans* (Figure 1). The *cis* pathway is important for the regenerative and protective functions of IL-6. In this pathway, the binding of IL-6 to its receptor, mIL-6R, and gp130 leads to the activation of the JAK (Janus kinase)/STAT3 (signal transducer and activator of transcription 3) pathway. Then, innate and acquired immunity is activated by this pathway, which leads to cytokine release syndrome. The *trans* pathway is also responsible for the pro-inflammatory activity of the cytokine [45]. Hence, Nrf2 and IL-6 may be introduced as therapeutic targets for COVID-19, especially for patients suffering from inflammatory problems. However, some trials have reported a lack of improvement in COVID-19 patient mortality upon IL-6 inhibitor treatment. According to Lowery et al., the most important reason for this paradox is that the pathogenic mechanisms of this viral disease are similar but not the same in all patients [46].

It should be noticed that COVID-19 patients in earlier stages of inflammatory diseases and GI symptoms, including malabsorption syndromes and IBD, are at a high risk of aggravating GI manifestation. It has been reported that the level of fecal calprotectin, a marker of bowel inflammation, is elevated in COVID-19 patients who suffer from diarrhea for more than 48 h [31]. It was found that ACE2 is highly expressed in inflammatory states, especially in IBD [47]. In IBD, age, inflammation, and disease location were identified as acute factors of the intestinal expression of ACE2. The role of ACE2 is controversial. On the one hand, it can regulate gut inflammation and diarrhea. However, on the other hand, its interaction with SARS-CoV-2 could lead to diarrhea [48]. Furthermore, it is known that ACE2 plays a significant role in gastric ulcer healing, which can be correlated with virus-mediated diarrhea [49]. Therefore, to provide a deep understanding of the aggravating symptoms in COVID-19 patients, especially those with gastric disorders, and determine therapeutic targets, ACE2-related signaling needs to be studied further in these patients.

#### 2.1.2. Antimicrobial Medications

Based on the available evidence, the use of different antimicrobial drugs such as antibiotics and antivirals can alter the gut flora and sensitize people to adverse GI effects [31]. Recent studies suggest that patients with altered gut microbiota might experience more severe COVID-19 symptoms. Diarrhea, nausea, and stomach pain are common adverse effects that can be induced by the various antivirals and antibiotics that are used in COVID-19 patient management [50]. However, through further research, whether these adverse effects have diagnostic value for COVID-19 could be determined [48]. Given that the use of antibiotic medications in COVID-19 patients could further change the digestive microbial flora, bacterial pneumonia treatment should only be initiated when clinical suspicion is high. However, the best antibiotic to use in COVID-19 patients with bacterial co-infections and GI symptoms remains unclear. In a review article published by Chedid et al. [49], data from 19 studies regarding antibiotic consumption in 2834 COVID-19 patients are analyzed. The use of antibiotic drugs occurred in 74% of patients. Only 17.6% of patients with COVID-19 who received antibiotics had secondary infections and 50% of the patients who received antibiotics were not severely ill, indicating the significant desire to initiate the use of antibiotics in mildly or moderately ill patients. Some retrospective studies have reported that antibiotic medication usage had no clear positive effect on mortality. However, detailed information on antibiotic treatment is lacking in most studies. Additional research to determine related manifestations for antibiotic use in COVID-19 patients is critical in light of the significant level of mortality associated with secondary infections in these patients, and the rising rate of antimicrobial resistance [51]. Some antivirals and antibiotics that have been used in COVID-19 treatment and reported on by several authors are listed in Table 1.

#### 2.1.3. Lung Flora Changes and Changes in the Ratio of Pathogenic Organisms

**Figure 2:**
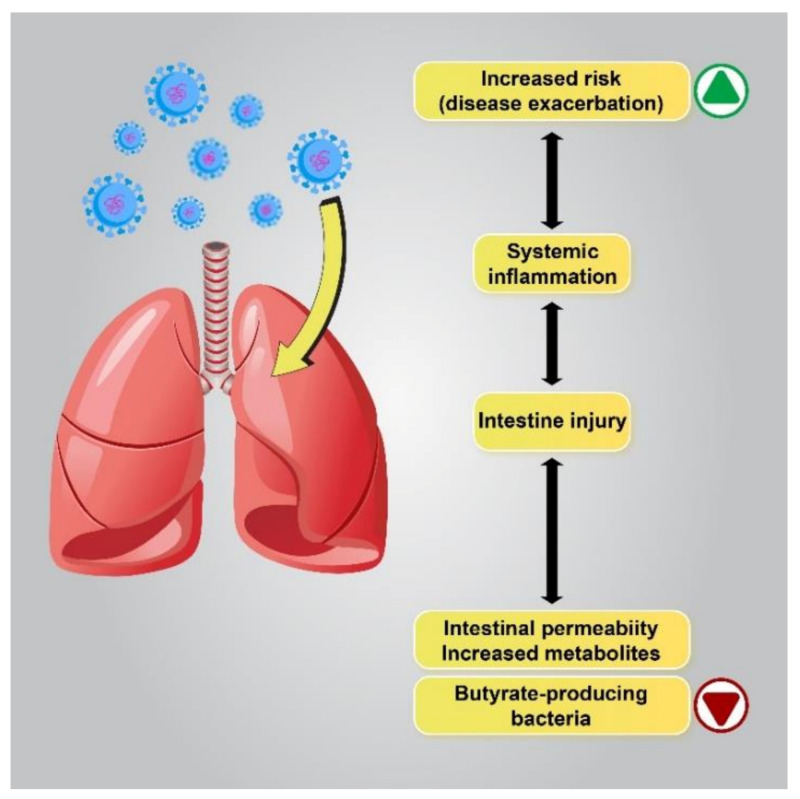
The correlation between flora changes and disease exacerbation. Patients with a lower gut abundance of butyrate-producing bacteria are more likely to develop viral lower respiratory tract infections. Dysbiosis alters metabolite production in the gut by enhancing the permeability of the intestines, which leads to the intensification of pre-existing lung diseases or an increase in the risk of respiratory diseases.

Based on recent reports on patients with respiratory problems, the “gut–lung” axis has been identified as a possible cause of GI disorders. Microbiota metabolites and the gut microbiota can regulate lung immunity through the lymphatic or circulatory systems. Several studies have reported a correlation between intestinal flora changes and disease exacerbation, and changes in the gut flora have been linked to lung disorders and infections of the respiratory tract [54,55,56]. Similarly, lung flora changes due to respiratory disorders in COVID-19 patients can be affected by the composition of the gut flora [31]. Immune reactions of the intestinal mucosal barrier, which protects the host against thousands of pathogens and environmental antigens, seem to correlate with pulmonary immune reactions [57,58,59] (Figure 2). 

Disrupted gut barrier integrity related to senility or underlying chronic disease, including obesity, diabetes, and hypertension, may be a main factor that permits the virus to gain access to the ACE2 receptor on the enterocytes and leak out of the digestive system to spread throughout the body. Therefore, if the gut immune barrier is disrupted, attacking microorganisms can enter the bloodstream or lungs and cause septicemia and acute respiratory distress syndrome (ARDS) [60]. Interestingly, several studies have demonstrated a close connection between COVID-19 severity and gut microbiota dysbiosis to be common. It has been reported that the abundance of beneficial bacteria belonging to the families Ruminococcaceae or Lachnospiraceae, the species Faecalibacterium prausnitzii, and the class Clostridia was reduced in COVID-19 patients. The class Clostridia is one of the major butyric acid-producing bacteria in the gut. The penetration of SARS-CoV-2 into the gut barrier may cause inflammation due to excessive immune responses that further increase gut permeability. In contrast, in the GI tract of healthy people or young children, who have a higher number of regulatory T cells (T_reg_ cells) due to their activation by butyrate, the virus may be contained in the digestive system without posing a considerable threat to the other organs of the body, eventually being excreted in the feces. Therefore, further investigation of the complex microbial interactions involving important butyrate-producing species is essential in order to understand its influence on human health and disease [61,62].

In a study conducted by Bradley et al., it was found that an abundance of segmented filamentous bacteria provokes the migration of Th17 cells to the lung, enhancing the autoimmune response and worsening pulmonary lesions [63].

Changes in the number of pathogens is another factor of microbiome alterations in people infected with SARS-CoV-2. Furthermore, altered flora also affects the ratio of pathogenic organisms, potentially leading to other infections such as *Clostridioides difficile* occurring in COVID-19 patients [31]. Foreign pathogenic organisms after infection with the virus and due to enterocyte absorption disorders increase the permeability of the intestinal barrier. Hence, theoretically, intestinal symptoms (such as diarrhea) demonstrate that the digestive system may be sensitive to SARS-CoV-2 infection [28]. 

#### 2.1.4. Enteral Nutrition

Given that no specific antiviral medications are available for COVID-19 patients, the immune strength of patients is crucial. Nutrition support is necessary to achieve adequate immune function. Oral feeding is a priority. When oral nutrition is difficult, enteral feeding should be considered as early as possible. In patients with acidosis, uncontrolled bleeding of the GI, uncontrolled hypoxemia, or other harmful conditions, delayed enteral feeding is suggested. Enteral feeding has been proposed for the maintenance of normal intestinal mucosal barrier function, as it prevents intestinal microbiome translocation and reduces the occurrence of infectious complications [64,65]. Enteral nutrition usage might alter the gut flora, which plays a significant role in maintaining GI homeostasis [31].

#### 2.1.5. Aberrant mTOR Activity and Deficit of ACE2

**Figure 3:**
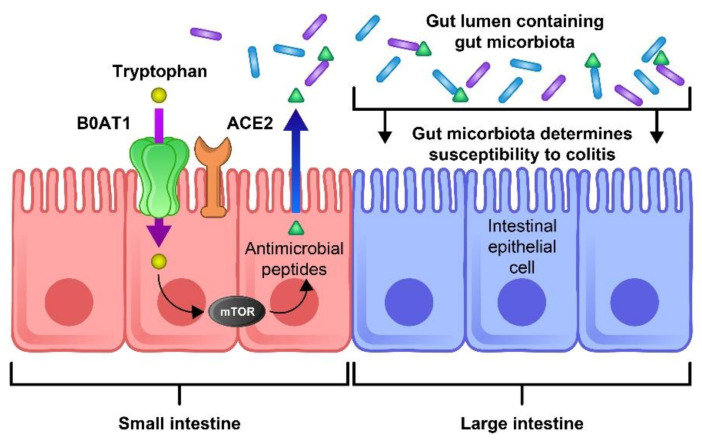
The role of ACE2 and mTOR activity in the expression of antimicrobial peptides and the composition of the gut flora. The ACE-2 receptor is essential for the surface expression of the neutral amino acid transporter B0AT1 in the small intestine. Tryptophan is mostly absorbed through the B0AT1/ACE2 pathway and activates the mTOR pathway, which regulates the expression of antimicrobial peptides. These peptides are important for maintaining an ideal microbiota in the large intestine. Thus, blocking this pathway by the binding of SARS-CoV-2 to the ACE-2 receptor could lead to inflammation, microbiota changes, and an increase in COVID-19 severity.

ACE-2 has been identified as a key regulator of neutral amino acid transporter in the small intestine and its deficit leads to the critical deficiency of local tryptophan homeostasis, which could alter the intestinal microbiome and an individual’s susceptibility to inflammation [66]. Consequently, impaired amino acid adsorption can lead to a low expression of antimicrobial peptides, leading to a change in the composition of the gut microbiota. ACE2 is essential for the surface expression of B0AT1 (the neutral amino acid transporter) in the small intestine. Dietary tryptophan is primarily absorbed via the transport pathway of B0AT1/ACE2 in the epithelial cells of the small intestinal; this results in mTOR pathway activation and the regulation of the expression of antimicrobial peptides. These peptides can adjust the gut microbiota composition [67]. The blocking of ACE2 and aberrant mTOR activity after SARS-COV-2 infection can molecularly explain how amino acid malnutrition results in intestinal inflammation and diarrhea. As SARS-CoV-2 S protein binds to the ACE2 receptor, blocking ACE2 leads to B0AT1 being blocked and thus tryptophan absorption being disturbed, leading to the alteration of the microbiota due to the aberrant secretion of antimicrobial peptides [67]. Hence, aberrant mTOR activity leads to a decreased expression of antimicrobial peptides from small intestinal Paneth cells (Figure 3). 

A recent study on the murine gut also reported that some microorganisms present in the intestine, such as *Bacteroidesthetaiotaomicron*, *Bacteroidesmassiliensis*, and *Bacteroidesdorei*, can downregulate ACE2 expression and inversely affect the SARS-CoV-2 load in a patient’s fecal samples [37].

## 3. Gastrointestinal Manifestations in SARS-CoV-2 and Outcome

The prevalence of GI symptoms in 12,797 patients from different studies is estimated to be about 20%, with no significant difference seen in terms of mortality rate [68]. The other issue caused by SARS-CoV-2 infection with GI manifestation is potential viral shedding. It has been reported that the pooled detection of the fecal samples of patients positive for viral RNA who were confirmed to have COVID-19 by nasopharyngeal swab testing or respiratory secretion analysis for PCR is 40.5% [69]. Severe diseases and increased CRP as predictors of high-risk infection have been observed in GI-presenting infected patients [70]. GI manifestations in patients with COVID-19 were found to be associated with a higher risk of ARDS, non-invasive mechanical ventilation, and tracheal intubation, but a similar link with mortality was not observed [71]. Patients with purely GI symptoms tended to have less severe disease and lower mortality rates compared to patients presenting with GI and respiratory effects [72]. In contrast to results found in the Chinese population, an Italian study reported better outcomes for patients presenting with GI symptoms and a related explanatory mechanism that considered a faster reduction in the viral load [73]. However, research conducted using a Bulgarian cohort has also indicated more severe disease in patients presenting with GI symptoms; this cohort makes up one third of all COVID-19 subjects investigated in this study [74]. Additionally, severe disease and fatal outcomes have been observed in the Indian COVID-19 patients presenting with GI symptoms [75]. According to the differences in outcomes of subjects presenting with GI symptoms in various populations worldwide, it is possible that the mechanism causing this is related to ACE polymorphism; its role in the recovery rate, mortality rate, and severity of illness of SARS-CoV-2 patients has been described [76].

## 4. The Correlation between Gastrointestinal and Neurological Symptoms Induced by SARS-CoV-2 

Although most reports on COVID-19 patients have focused on the failure of the respiratory system and its symptoms, many researchers have also reported on intestinal and neural disorders induced by SARS-CoV-2. Nevertheless, the mechanisms of neurological or GI symptoms in COVID-19 patients have not been identified with any certainty. Interestingly, some symptoms, including vomiting and nausea, could be signs of either digestive system or nervous system disorders. SARS-CoV-2 causes central and peripheral nervous system symptoms, such as insanity, neck resistance, hypogeusia, hyposmia/anosmia, headache, nausea, vomiting, and psychiatric and psychological symptoms, in a large percentage of patients with COVID-19 [77,78,79]. Combining these symptoms with headache or high intracranial pressure may be a sign of central nervous system (CNS) infection. 

**Figure 4:**
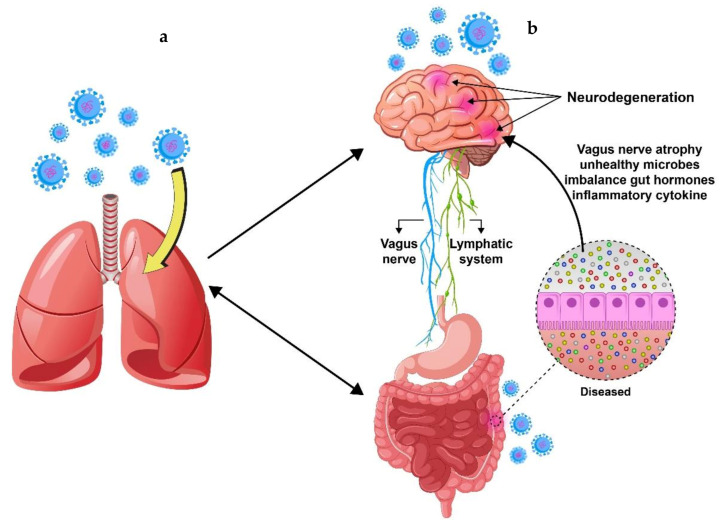
Cross-talk between the lungs (**a**), brain, and gastrointestinal tract (**b**). The metabolites produced by gut microbes not only impact GI immunity but also affect distal organs such as the lungs and brain. The gut and lungs are intricately linked organs that influence each others’ homeostasis through bi-directional cross-talk (termed the gut–lung axis). SARS-CoV-2, by altering the lung microbial community, can influence the composition of the gut microbiota. Similarly, when the composition and diversity of the gut microbiota are changed, the gut microbiota can regulate the lung immune function through the lymphatic or circulatory systems. Additionally, alterations in the intestinal microbiota can lead to CNS disorders via interactions through the gut–brain axis.

Thus, it seems that there is a complex cross-talk between the lungs, brain, and gastrointestinal tract during severe COVID-19 illness (Figure 4). Due to this, physicians should pay more attention to the common symptoms [80]. While there are plenty of theories on the route by which neuro-invasion occurs, histological evidence and detailed information on neurological symptoms and the connection between headache, nausea, and vomiting or intracranial hypertension have been neglected [81].

The precise documentation of GI signs and neurological, electrophysiological, and postmortem investigations of brain tissue in COVID-19 patients with GI symptoms could help us to clarify how SARS-CoV-2 causes gastrointestinal and neurological manifestations. More recently, a relation between intestinal inflammation (by fecal calprotectin determination) and the incidence of fecal SARS-CoV-2 RNA in hospitalized individuals has been reported [82]. Pro-inflammatory intermediates in the GI tract can form a path to the brain through the vascular or lymphatic system [83,84,85]. Additionally, virus-welded gut inflammation may trigger cognitive functions via the vagal nerve (Figure 4). 

Additionally, Esposito et al. [86] have proposed that the infected enteric nervous system (ENS) could contribute to the worsening of cytokine storms elicited by COVID-19. This has been supported by extensive investigations of the immunological properties of enteric glial cells [87]. Surprisingly, Deffner et al. [81], using constitutive histological evidence, have observed that cellular entry routes, including ACE2 and TMPRSS2, are highly expressed by enteric neurons and glial cells of the small and large intestine, as well as the blood–brain barrier and choroid plexus epithelial cells. This means that these cells meet the molecular demands for virus entrance [81]. 

From a systemic view, it seems that there are neural and hormonal links between the lateral hypothalamic nuclei and the GI tract that lead to temporal correlation between the symptoms of these two systems. Additionally, an increasing amount of evidence supports the hypothesis that the gut may be the “entrance door” and that the temporal correlation between gastrointestinal and neurological symptoms indicates the lymph vessels around the GI tract, vascular system, or ENS (gut–brain axis) to be the most likely entry route for SARS-CoV-2 to the brain and CNS [7,88].

The ENS is strictly controlled, with early gastric cancers (EGCs) and the gut epithelium collaborating in a neuro-epithelial unit that is crucial for gut homeostasis. Major histocompatibility complex II, which is expressed by EGCs, mainly responds to harmful stimuli via the Toll-like receptors-2 and -4, regulates the neuro–immune axis, and defends the host against gut pathogens. Furthermore, the activation of EGCs is attended by a vast release of IL-6 and other inflammatory intermediates that could result in acute respiratory discomfort, as observed in the SARS-CoV-2 induced cytokine storms experienced by COVID-19 patients [86]. 

## 5. Effect of SARS-CoV-2 Spike Protein on Endothelial Cells and Blood–Brain Barrier

According to the findings published by Buzhdygan et al. [89], SARS-CoV-2 spike proteins stimulate a pro-inflammatory response in brain endothelial cells that may have a negative effect on blood–brain barrier integrity and disrupt its function. Altered function of the blood–brain barrier greatly increases the possibility of neuro-invasion by this virus, offering an explanation for the risk of neurological damage in COVID-19 patients. These results are the first reports on the neurological consequences experienced by COVID-19 patients caused by the direct impact of the SARS-CoV-2 spike protein on the brain endothelial cells [89]. 

Furthermore, it can be speculated that one of the reasons for the neurological complications seen in COVID-19 patients is an interaction of the spike protein, or, more accurately, spike S1 protein receptor binding domain (SARS-CoV-2 S1 RBD), with amyloidogenic proteins in the brain. It has recently been shown via molecular docking that this domain can interact with β-amyloid peptide, α-synuclein, tau protein, and prion protein [90]. However, these results have not yet been confirmed on isolated proteins or their fragments. In the laboratory of Prof. Muronetz, studies on the interaction of SARS-CoV-2 S1 RBD with α-synuclein and prion protein have been carried out using the SPR (surface plasmon resonance) method. In addition, studies on the effect of SARS-CoV-2 S1 RBD on the amyloid transformation of α-synuclein and prion have been conducted through the detection of the beta-sheet structures of Congo red and the fluorescence of thioflavin T after staining with a specific dye. Moreover, the influence of cinnamic acid derivatives on these processes needs to be tested, since we showed previously that these compounds selectively interact with prion protein [91] as well as α-synuclein [92], preventing their amyloid transformation. We think that the cinnamic acid derivatives interacting with amyloidogenic proteins may change their conformation and thus prevent binding to SARS-CoV-2 S1 RBD. It is possible that these substances, which include some natural compounds present in food products, can restrict the development of neurodegenerative consequences of COVID-19 infection.

## 6. Hepatic Derangements, Viral Hepatitis, and COVID-19 

Hepatic problems associated with COVID-19, including liver enzyme abnormalities, have been observed by the clinicians involved in the management of the disease at an alarming rate. Nearly 50% of patients have been observed to experience complications of COVID-19 and thus additional impairments [93]. Among such augmented biochemical and clinical derangements, the appearance of disproportionate levels of liver enzymes has gradually been established as a serious cause for concern for clinicians treating COVID-19. The cytopathy of hepatocytes by SARS-CoV-2 infection has been observed. However, to what extent this contributes to liver impairment remains debatable. In an interesting study carried out by Wang et al. on 156 patients with COVID-19 where the clinical features between abnormal and normal liver enzyme groups were compared, the authors concluded that the direct SARS-CoV-2 infection of liver cells had significantly contributed to hepatic impairment among patients with COVID-19. In this study, biopsied liver tissues were subjected to histological, ultrastructural, and immunohistochemical examination [93].

At the same time, it is believed that SARS-CoV-2 has a lesser likelihood of causing liver infection because of the diminished expression of ACE2 in liver cells. However, surprisingly, Wang et al. reported an abundance of SARS-CoV-2 viral particles in hepatocytes [93]. These disagreements regarding the systemic presence of SARS-CoV-2 and tissue-specific ACE2 expression could have multiple dimensions of arguments. Several interesting studies have highlighted the discordances of ACE2 expression in multiple organs targeted by SARS-CoV-2 [94,95]. For example, despite no ACE2 being detected in the colonic epithelium, virus replication was confirmed, while endothelial cells harboring ACE2 did not show the virus infection [96]. Taking these observations into account, research exploring the alternative to ACE2 receptors or the existence of co-receptors needs to be carried out. However, the exaggerated expression of ACE2 in hepatocytes as a consequence of sensing the viral entry may also not be ruled out. COVID-19-related complications pertaining to deranged liver enzymes have also been attributed to additional factors, such as the development of hypoxic-ischemic liver injury, the possibility of sepsis, and drug-induced liver injury (DILI) [97,98]. Furthermore, it is also known that the first few weeks after antibiotic therapy have the potential to cause hepatotoxicity related to the substantial elevation of liver enzymes. To be more precise, both SARS-CoV-2 infection and drugs might result in liver steatosis [99,100,101]. A close monitoring of viral clearance in the liver and, subsequently, of the long-term outcome of COVID-19, are hence essential.

Liver co-morbidities are known in around 2–11% of COVID-19 patients, where dysregulated levels of alanine amino transferase (ALT) and aspartate amino transferase (AST) have been reported in some 14–35% of cases, coinciding with disease progression [102,103,104]. In the course of HBV infection, viral clearance and liver inflammation have been attributed to the role played by HBV-specific T lymphocytes. As a consequence, viral persistence might be related to qualitative and quantitative abnormalities in HBV-specific T cell responses [105]. Clinicians and researchers have continuously sought to determine whether the existence of HBV, the spontaneous resolution of viral hepatitis, or past exposure to HBV could affect SARS-CoV-2 infection and outcomes. Whether SARS-CoV-2 infection leads to the further deterioration of liver function among patients with pre-existing HBV infection has become a recent matter of concern for clinicians and researchers. Few studies have indicated the characteristics of liver function in patients with SARS-CoV-2 and chronic HBV co-infection. In a recent study conducted by Xiaojing Zou et al. on 105 patients with SARS-CoV-2 and chronic HBV co-infection, elevated levels of liver enzymes were observed in several patients, with ALT elevation seen in 21% and AST elevation seen in 27.6%. This interesting and quite relevant study demonstrates that the incidence of severe COVID-19 was significantly higher in patients with liver injury, causing acute-on-chronic liver failure, acute cardiac injury, and shock, which were all seen more frequently among patients with liver injury. Mortality was significantly higher in individuals with liver injury. It was concluded that liver injury in patients with SARS-CoV-2 and chronic HBV co-infection was associated with the severity and poor prognosis of the disease [105].

On the other hand, Liping Chen et al. retrospectively compared 20 COVID-19 patients with HBV co-infection with 306 COVID-19 patients without HBV infection, and no differences were shown in the liver function parameters. The authors also claimed that there were no significant differences in the discharge rate and length of stay between two groups; on this basis, they inferred that no evidence was found that SARS-CoV-2 /HBV co-infection could aggravate liver injury or extend the duration of hospitalization. Data were collected in this retrospective study from a COVID-19-designated hospital in Shanghai, China, from January 2020 to 24 February 2020, with follow-up until 29 February 2020 [106]. Limitations associated with the retrospective collection of data, lack of anti-HBV antiviral therapy, and significantly lower number of HBV-co-infected patients (only 20 compared with 306 patients) should not be ignored in this study. Another study on a similar topic conducted among 50 SARS-CoV-2 and HBV-co-infected patients, 56 SARS-CoV-2 mono-infected patients, 57 HBeAg negative chronic hepatitis B controls, and 57 healthy controls from Renmin Hospital of Wuhan University demonstrated that SARS-CoV-2 and HBV co-infection did not significantly affect the outcome of COVID-19. This group of researchers also included cytokine measurements of IFN-γ, TNF-α, IL-2, IL-4, IL-6, and IL-10. They also measured the T-cell, B-cell, and NK-cell counts. At the onset of COVID-19, SARS-CoV-2 and HBV-coinfected patients showed more severe monocytopenia and thrombocytopenia as well as more disturbing hepatic function in albumin production and lipid metabolism. Most of this disarrangement could be reversed after recovery from COVID-19. This finding could be attributed to the limited role of pre-existing HBV infection in the final outcome of COVID-19 [107].

Researchers remain very curious regarding the interactions between SARS-CoV-2 and HBV in cases of co-infection. However, until now there has been no concrete evidence shedding light on this phenomenon due to the lack of availability of a considerable number of HBsAg-positive patients in any single study. Therefore, whatever findings have been noted can only point towards glimpses of possibilities. In a recent study by Yu et al., the authors tried to address the same issue by enrolling COVID-19 patients from the previous cohort and classifying them into two groups (7 being HBsAg positive and 60 being HBsAg negative). However, in order to navigate the interaction phenomenon, they analyzed the association of SARS-CoV-2 and HBV-related markers. During the acute course of SARS-CoV-2 infection, markers of HBV replication did not substantially fluctuate. Furthermore, the co-infection of HBV did not extend the viral shedding cycle or incubation periods of SARS-CoV-2; hence, they concluded that the effects of SARS-CoV-2 on the dynamics of chronic HBV infection were not visible and hence could not be related to the source of HBV reactivation, at least in the seven patients with chronic HBV that they studied [108]. However, the number of HBsAg-positive patients was too low to address this issue with any conviction. Yong Lin et al. [109] reported that inactive HBV carriers of SARS-CoV-2 infection are at a higher risk of abnormal liver function tests. They found that the elevated liver injury induced by SARS-CoV-2 and HBV infection was of the hepatocyte type rather than the colangiocyte type. For this, they noted that the inflammatory response, including abnormal lactate dehydrogenase, D-dimer, and interlukin-6 production, may contribute to injury following SARS-CoV-2 infection. These findings were obtained from a retrospective study of 133 hospitalized patients confirmed to have mild COVID-19, including 116 patients with COVID-19 with the negative serum hepatitis B antigen and only 17 HBV-inactivated carriers with COVID-19. No significant differences between groups in terms of the discharge rate or duration of hospitalization were reported. Nevertheless, they reached the conclusion that SARS-CoV-2 and HBV co-infection worsened the liver function of patients with COVID-19 [109]. Jiaye Liu et al. [110], in a recent study, argued that COVID-19 patients co-infected with chronic HBV could experience a risk of reactivation of hepatitis B in an observation of longitudinal changes in liver function among COVID-19 patients with pre-existing chronic HBV infection. They recommended monitoring the liver functions of COVID-19 patients as well as the HBV DNA levels for those co-infected with HBV during the whole course of the disease [110]. Table 2 includes a summary of studies on COVID-19 patients with pre-existing Hepatitis B virus (HBV) infection.

As treatment for COVID-19 may include immunosuppressive therapies, such as IL-6 receptor antagonists and corticosteroids, the risk of HBV reactivation in severe COVID-19 patients with resolved HBV infection undergoing immunosuppressive therapy is definitely a matter that needs further investigation. In such a unique study, HBV reactivation prophylaxis with entecavir has been observed. The clinicians involved in this study recommended entecavir prophylaxis to 61 out of 600 COVID-19 patients depending upon the inclusion criteria defined in HBV-co-infected cases. However, only 38 out of 61 patients received entecavir. Though the number of dropouts in this prospective study was quite high, through a meticulous analysis they reached the interesting conclusion that the risk of HBV reactivation in patients with severe COVID-19 and resolved HBV infection undergoing immunosuppressive treatment was low. The immunosuppressive drug used the most was tocilizumab [111]. Based on the outcome of this extensive study, the authors recommended that if a systematic follow-up after hospital discharge is infeasible without anti-HBs, a short course of anti-viral prophylaxis may be a safe option and may contribute to a low risk of hepatitis B reactivation in patients with severe COVID-19 who have received immunosuppressive therapy.

For serious viral hepatitis, especially for HBV infection-related co-morbidities in COVID-19 patients, it is gradually becoming important to know the risk of HBV reactivation. Moreover, therapeutic interventions related to the use of immunosuppressive drugs such as tocilizumab and corticosteroids to treat COVID-19 should be decided on carefully, considering the possibility of the reactivation of pre-existing HBV infection. Among SARS-VCoV-2- and HBV-co-infected patients, prophylactic measures for the prevention of HBV reactivation are important issues for which further investigations are warranted [112,113].

## 7. Conclusions

As mentioned, SARS-CoV-2 enters the respiratory system and systemic circulation and accesses other systems, including the liver, GI, and CNS. The main mechanism of the entry of the virus into cells is mediated by the ACE-2 receptor, which is widely expressed in these systems. Patients with COVID-19 experience changes in their gut microbiota, dysbiosis, and gastrointestinal symptoms as well as changes in their lung microbiota. It seems that there is an inverse correlation between the abundance of beneficial gut bacteria and disease severity. Additionally, there is crosstalk between the lungs, brain, and gut; thus, the microbiome alterations, dysbiosis, and exacerbated inflammation induced by COVID-19 could affect these organs and increase the risk of GI problems, mood disorders, and neurodegenerative diseases. Additionally, we mentioned that cytokine storms are a major cause of worsening conditions in COVID-19 patients, including those with gastrointestinal disorders and severe COVID-19. Thus, a potential strategy to prevent severe disease may be the recovery of proper intestinal flora composition and the suppression of cytokine storms. We hope that this review will provide up-to-date information on the efforts to discover potential pharmacotherapeutic goals for the treatment of these symptoms in the future.

## Figures and Tables

**Table 1:** Some antiviral and antibiotics that are used in COVID-19 treatment, as reported by Chedid 2021 [51], Bagheri 2021 [52], and Frediansyah 2021 [53].

Drug/Drug Class	Therapeutic Category	Reference
Aminoglycosides	Antibiotic	Bagheri 2021
Azithromycin	Antibiotic	Bagheri 2021, Chedid 2021
Moxifloxacin	Antibiotic	Chedid 2021
Ceftriaxone	Antibiotic	Chedid 2021
Cephalosporin	Antibiotic	Chedid 2021
Quinolones	Antibiotic	Chedid 2021
Clarithromycin	Antibiotic	Chedid 2021
Ceftriaxone	Antibiotic	Chedid 2021
Tigecycline	Antibiotic	Chedid 2021
Cefoperazone	Antibiotic	Chedid 2021
Umifenovir	Antiviral	Frediansyah 2021
Lopinavir	Antiviral	Frediansyah 2021
Darunavir	Antiviral	Frediansyah 2021
Atazanavir	Antiviral	Frediansyah 2021
Saquinavir	Antiviral	Frediansyah 2021
Emtricitabine	Antiviral	Frediansyah 2021
Azvudine	Antiviral	Frediansyah 2021
Remdesivir	Antiviral	Frediansyah 2021
Favipiravir	Antiviral	Frediansyah 2021
Ribavirin	Antiviral	Frediansyah 2021
Sofosbuvir	Antiviral	Frediansyah 2021
Oseltamivir	Antiviral	Frediansyah 2021

**Table 2:** Summary of studies on COVID-19 patients with pre-existing hepatitis B virus (HBV) infection.

Authors	Study Design	Salient Findings/Observations	Whether Associated with the Final Outcome of COVID-19
Zou X. et al. [105]	105 patients with SARS-CoV-2 and chronic HBV co-infection were studied to determine their biochemical and clinical outcomes.	Biochemical parameters (ALT, AST, total bilirubin, AFP) increased significantly during hospitalization. 4 of 14 patients who developed liver injury rapidly progressed to acute-on-chronic liver failure. The proportion of severe COVID-19 was higher in patients with liver injury (*n* = 14) (*p* = 0.42) including acute-on-chronic liver failure and, acute cardiac injury (*p* < 0.05). Mortality higher among patients with liver injury (*p* = 0.004)	Yes
Chen L. et al. [106]	Clinical study to evaluate whether SARS-CoV-2/HBV co-infection could influence liver function and disease outcome among 20 patients with HBV co-infection vs. 306 patients without HBV co-infection.	No differences in the level of liver functions parameter. No significant differences in terms for the discharge rate and length of stay between the two groups.	No
Liu R. et al. [107]	50 SARS-CoV-2 and HBV co-infected patients, 56 SARS-CoV-2 mono-infected patients, 57 HBeAg negative chronic HBV patient controls and 57 healthy controls. Serum biochemical parameters and cytokines were assessed. T cell, B cell and NK cell counts were measured.	SARS-CoV-2 and HBV co-infection did not significantly affect the outcome of COVID-19. Most of the disarrangement including severe monocytopenia and thrombocytopenia as well as disturbed hepatic function with respect to albumin production and lipid metabolism was reversed after recovery from COVID-19.	No
Yu R. et al. [108]	SARS-CoV-2 infected patients with HBsAg +ve serology (*n* = 7) and with HbsAg –ve serology (*n* = 60) were studied.	SARS-CoV-2 did not affect the dynamics of chronic HBV infection and was not found to be the source of HBV reactivation. Markers of HBV replication did not extensively fluctuate during SARS-CoV-2 infection.	No
Lin Y. et al. [109]	116 COVID-19 patients with HBV negative serology and 17 COVID-19 patients with HBV serology but as inactive carriers were studied.	Though there were significant differences for the discharge rate or duration of hospitalization, SARS-CoV-2 and HBV co-infection among 17 patients were found to have exacerbated liver function. COVID-19 with inactive HBV carriers with SARS-CoV-2 co-infection were at higher risk of abnormal liver functions.	Undecided whether the final outcome of COVID-19 was influenced.
Liu J. et al. [110]	Included 21 vs. 326 as with vs. without chronic HBV infection. However, with the help of the Propensity Score Method (PSM) final inclusion was restricted to 20 vs. 57 for the HBV group and non-HBV group, respectively.	All the 71 patients included after applying PSM, achieved SARS-CoV-2 clearance. No significant difference between the two groups showing progression to severe COVID-19. Longitudinal changes in biochemical parameters between the two groups were not significantly different. However, 3 patients in the HBV group experienced reactivation of HBV.	No (however, the authors suspect HBV reactivation and suggest monitoring the liver functions as well as HBV DNA levels of COVID-19 patients during the whole disease course.)

## Data Availability

No new data were created or analyzed in this study. Data sharing does not apply to this article.
